# In vitro biomechanical testing of the 3.5 mm LCP in torsion: a comparison of unicortical locking to bicortical nonlocking screws placed nearest the fracture gap

**DOI:** 10.1186/s13104-017-3102-y

**Published:** 2017-12-27

**Authors:** Alex A. Padron, John R. Owen, Jennifer S. Wayne, Sevima A. Aktay, Roy F. Barnes

**Affiliations:** 1 Virginia Veterinary Surgical Associates a BluePearl® Partner, 5918 W. Broad Street, Richmond, VA 23230 USA; 2 Departments of Orthopaedic Surgery and Biomedical Engineering, Orthopaedic Research Laboratory, 325 McGuire Annex 1112 E. Clay Street, Virginia Commonwealth University, Richmond, VA 23298-0694 USA

**Keywords:** Biomechanical testing, Torsion, Locking compression plate

## Abstract

**Objective:**

This biomechanical study compared the torsional strength and stiffness of a locking compression plate with all locking versus nonlocking screws and examined the effect of placing a locking unicortical or nonlocking bicortical screw nearest the fracture gap in a synthetic bone model.

**Results:**

Synthetic bone models simulating a diaphyseal fracture without anatomic reduction were tested using four screw configurations: all bicortical locking (ABL), all bicortical nonlocking (ABN), a hybrid construct with a bicortical nonlocking screw nearest the fracture gap (BN), and a unicortical locking screw placed nearest the fracture gap (UL). Torsional stiffness, rotation and torque at failure were compared via ANOVA and post hoc pairwise comparisons (p < 0.05). ABN and BN had the highest stiffness (p < 0.01) with ABL greater than UL (p < 0.01). Rotation at failure was greatest for ABL (p < 0.01) with UL greater than ABN (p < 0.05). Unicortical locking screws nearest the fracture gap decreased stiffness, without significantly affecting torque or rotation at failure. Construct stiffness was found to exist in a very narrow range of 0.9–1.2 N m/deg with standard deviations of 0.1 N m/deg in all cases. The results of this study support the use of nonlocking screws in a hybrid construct to increase torsional stiffness.

**Electronic supplementary material:**

The online version of this article (10.1186/s13104-017-3102-y) contains supplementary material, which is available to authorized users.

## Introduction

In 2001 the locking compression plate (LCP) (Synthes^®^, West Chester, PA, USA) was created which merged the geometry of the limited contact dynamic compression plate (LC-DCP) (Synthes^®^, West Chester, PA, USA) with a conical threaded hole, resulting in the so called ‘combi-hole’ which allows the surgeon to choose between a dynamic compression plate, fixed angle locking construct or a combination of locking and nonlocking screws resulting in a ‘hybrid’ construct [1]. Only a few studies have evaluated the use of ‘hybrid’ constructs where locking and nonlocking screws are used in the same plate [2–7]. These studies used bicortical screws in their constructs.

A recent study comparing different screw combinations in hybrid constructs concluded that hybrid constructs were at least as stiff and strong as all locked constructs. This study however did not demonstrate a statistically significant difference in torsional strength between the all locked and hybrid constructs. The authors went on to suggest that perhaps a difference would be detected if monocortical fixation was compared [7].

To our knowledge no studies have evaluated the biomechanical differences between placing a monocortical locking screw nearest the fracture gap versus a bicortical nonlocking screw in a hybrid construct. Bicortical screw placement may be inherently advantageous to monocortical screw placement due to the increased working length of the screw but there are clinical scenarios where the placement of a bicortical screw is not feasible due to the risk of entering an articular surface, the presence of an intramedullary rod, or risk of losing primary reduction by entering a fissure or fracture line on the trans cortex. In these scenarios a surgeon must choose between either placing a monocortical locking screw, leaving the screw hole empty, or attempting bicortical engagement with a nonlocking screw which can be angled in a particular direction. The objective of this study was to compare the torsional strength and stiffness of the 3.5 mm LCP with all locking versus nonlocking screws and the effect of placing a locking unicortical or nonlocking bicortical screw in a diaphyseal fracture gap model. Our hypothesis was that the bicortical nonlocking screw in a hybrid construct would provide more torsional resistance than a unicortical locking screw when placed nearest the fracture gap.

## Main text

### Methods

#### Bone model

Fourth generation Sawbones (SKU:3403-4 Pacific Research Laboratories, Inc., Vashon Island, WA, USA) with 3 mm wall thickness and 20 mm outer diameter were divided into four groups and used to simulate bone segments. This synthetic bone model has been previously validated and used in similar studies [8, 9].

#### Plates and screws

**Fig. 1:**
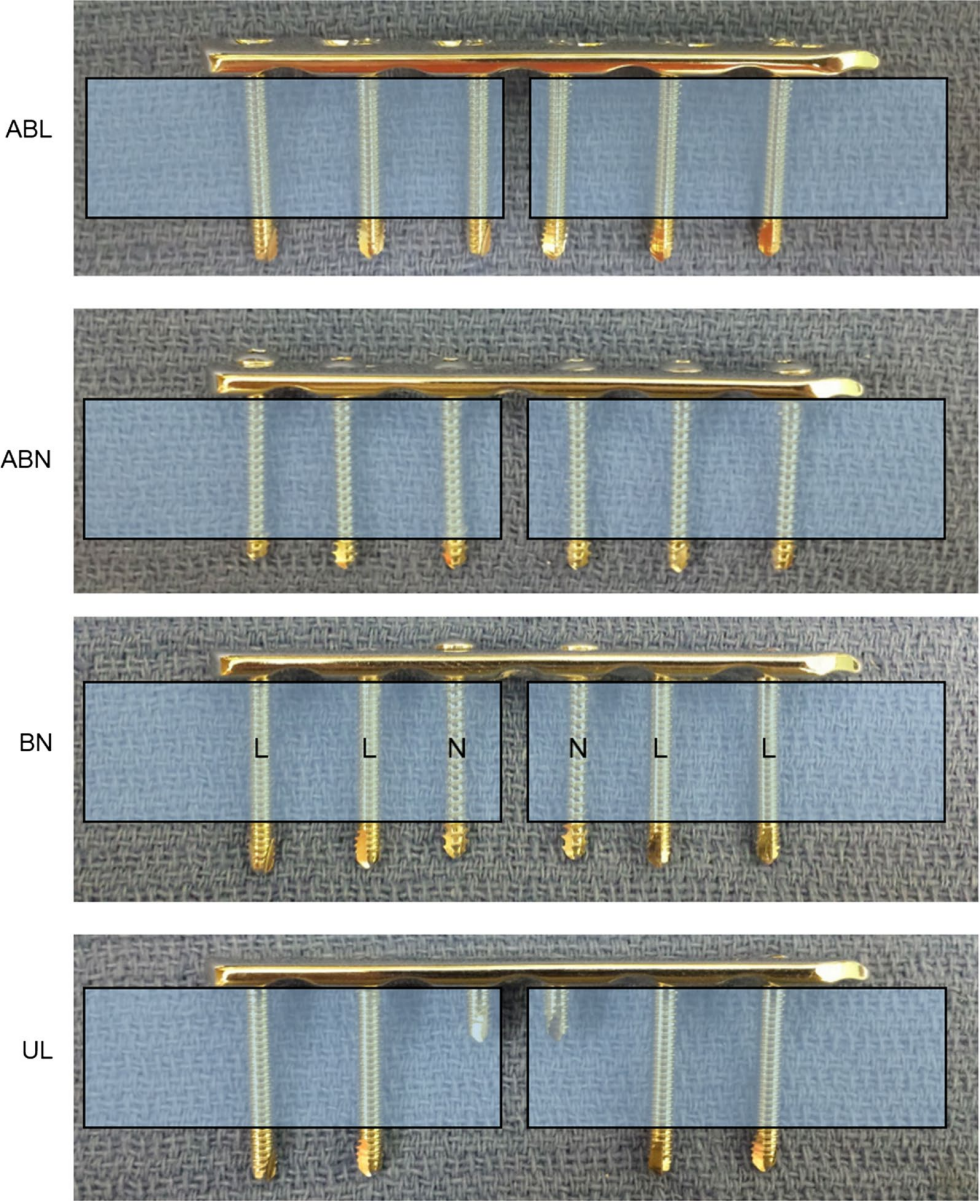
Screw configurations. Schematic representation of the four screw configurations from top to bottom: all bicortical locking (ABL), all bicortical nonlocking (ABN), single bicortical nonlocking screw nearest fracture gap (BN), single unicortical locking screw nearest fracture gap (UL)

Forty 6-hole 3.5 mm LCP (VP 4041.06 Synthes^®^, West Chester, PA, USA) were tested using four different screw configurations of locking bicortical and unicortical screws and nonlocking cortical screws. The first group termed ‘all bicortical locking’ (ABL n = 10) was comprised of all 3.5 mm × 26 mm length bicortical locking screws (VS303.026 Synthes^®^, West Chester, PA, USA). The second construct termed ‘all bicortical nonlocking’ (ABN n = 10) was comprised of all 3.5 mm × 26 mm length bicortical nonlocking screws (VS302.026 Synthes^®^, West Chester, PA, USA). The third construct was a “hybrid” construct termed ‘bicortical nonlocking’ (BN n = 10) was comprised of a single 3.5 mm × 26 mm length bicortical nonlocking screw placed nearest the fracture gap with two 3.5 mm × 26 mm length bicortical locking screws on either side of each nonlocking screw. The fourth construct termed ‘unicortical locking’ (UL n = 10) was comprised of one 3.5 mm × 10 mm length unicortical locking screw (VS303.010 Synthes^®^, West Chester, PA, USA) placed nearest the fracture gap with two 3.5 mm × 26 mm length bicortical locking screws on either side of each unicortical locking screw. See (Fig. 1).

#### Construct assembly

The SFE bone model was cut into 250 mm long segments. Each bone plate was centered on the 250 mm cylinder and secured using stainless steel self-tapping cortical bone screws using standard AO ASIF technique. All nonlocked screws were placed before any locked screws. The screw nearest the simulated fracture site was inserted first followed by the symmetrically positioned screw on the opposite side of the fracture. Insertion of the screws was alternated working outward toward the ends of the construct. Pilot holes for the locking screws were drilled using a 2.8 mm drill bit through a drill guide (312.618 Synthes^®^, West Chester, PA, USA) that locked into the plate. Pilot holes for the nonlocking screws were drilled using a 2.5 mm drill bit and drill guide (322.32 Synthes^®^, West Chester, PA, USA) placed in the neutral position of the compression hole.

A 1 mm osteotomy gap was created at the center of each construct using a miter box saw with a 1 mm blade width. All screws were fully inserted and tightened in the same order they were placed. All screws were tightened to 2.6 Nm of torque with a calibrated microtorque screwdriver (MT50AFH; Mountz, San Jose, California) immediately prior to testing.

#### Biomechanical testing

**Fig. 2:**
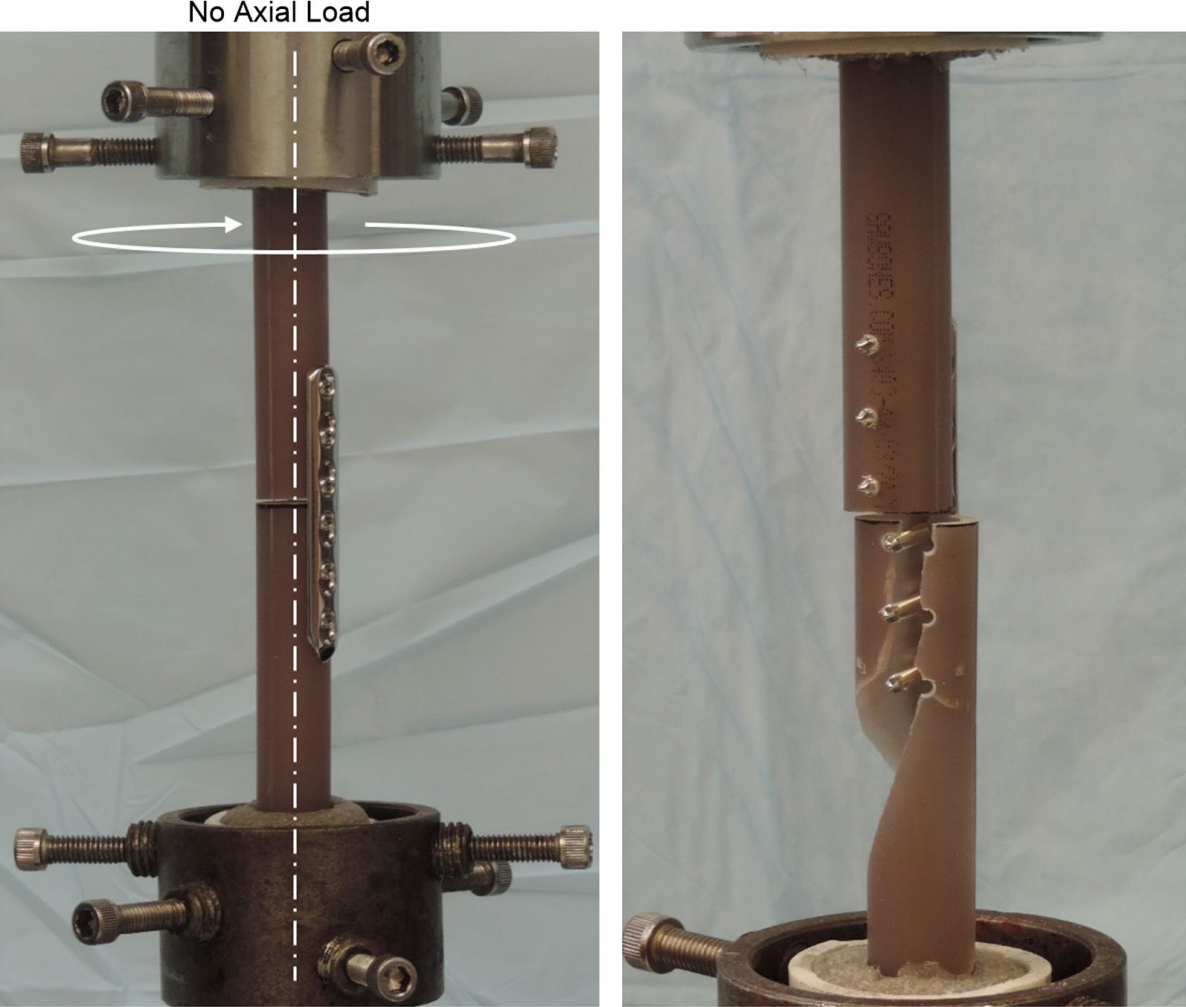
Construct setup. Testing set up for applying a torsional load under zero axial load and center of rotation (left). Photograph of synthetic bone fracture in a typical spiral fracture configuration propagating through all distal screw holes (right)

The ends of each cylinder were secured in a 40 mm × 76 mm polyvinyl chloride (PVC) pipe using polymethylmethacrylate (Technovit J0061; Jorgenssen Laboratories Inc, Loveland, Colorado) and two 1/8 inch transfixing pins drilled through the cylinders and PVC pipe. The cylinders were then placed in a model 1321 Instron biaxial servohydraulic testing machine (Instron Corp., Canton, Massachusetts) outfitted with a TestStar II system (MTS TestStar™ II, MTS Corp., Eden Prairie, MN) for digital control and data acquisition (Fig. 2). Constructs were tested in angular displacement control and zero axial load was applied. Torque and displacement data were recorded at a rate of 20 Hz. Constructs were tested in a single cycle to catastrophic failure in torsion at a rate of one degree per second.

#### Data analysis

All data was reported as mean ± standard deviations (mean ± SD). Torque versus angle curves were generated and the peak torque (strength in N m) and peak angular displacement (rotation in degrees) at failure were determined. The linear slope of the torque versus angular displacement curve between 0 and 10 N m of torque was analyzed using linear regression to calculate torsional stiffness (N m/deg). Failure was defined as an abrupt decrease in the load due to fracture of the synthetic bone. A description of the failure mode (fracture configuration) and a qualitative assessment of the plastic deformation of plates and screws were recorded at the end of each test. An analysis of variance was performed followed by Tukey post hoc pairwise comparisons using statistical analysis software (SAS^®^ Enterprise guide 5.1^®^, Cary, NC). Statistical significance was set at p < 0.05.

### Results

#### Peak torque to failure

**Fig. 3:**
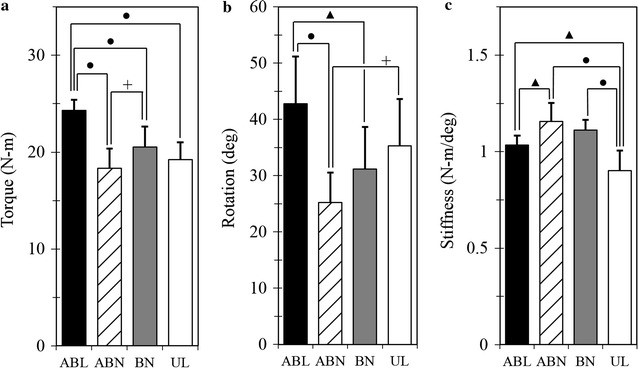
**a** Peak torque. Peak torque values at catastrophic failure (mean ± SD). The ABL construct had a significantly higher peak torque than all other constructs (ABL > ABN, BN, UN, p ≤ 0.0002). The BN construct had a significantly higher peak torque than the ABN construct (p = 0.044). The constructs with the lowest peak torque to failure were ABN and the UL construct though no significant difference was noted between the two groups (p = 0.375). Significant differences indicated by connecting lines are denoted by ^**∙**^p < 0.001, ^▲^p < 0.01, and ^**+**^p < 0.05. **b** Angular displacement. Peak rotation/angular displacement at catastrophic failure (mean ± SD). The ABL construct had a significantly higher angular displacement at peak torque than the BN and ABN constructs (p ≤ 0.007). The UL construct had a significantly higher angular displacement at peak torque than the ABN construct (p = 0.023). No statistically significant difference was noted between the UL and BN constructs (p = 0.606). Additionally, no significant difference was noted between the ABL and UL (p = 0.132) constructs in angular displacement at peak torque. Significant differences indicated by connecting lines are denoted by ^**∙**^p < 0.001, ^▲^p < 0.01, and ^**+**^p < 0.05. **c** Torsional stiffness. Torsional stiffness of the 4 constructs (mean ± SD). The ABN construct was significantly stiffer than the ABL and UL constructs (p ≤ 0.007). No significant difference was detected between the ABN and BN constructs in torsional stiffness (p = 0.588). Additionally, no significant difference was detected between the ABL and BN constructs (p = 0.144). The UL construct had significantly less torsional stiffness than all other constructs (p ≤ 0.004). Significant differences indicated by connecting lines are denoted by ^**∙**^p < 0.001, ^▲^p < 0.01, and ^**+**^p < 0.05

Peak torque to failure was greatest for the ABL construct (ABL > ABN, BN, UN, p ≤ 0.0002). The BN construct had a significantly higher peak torque than the ABN construct (p = 0.0440). The constructs with the lowest peak torque to failure were ABN followed by the UL construct though no significant difference was noted between the two groups (p > 0.37). The mean ± SD torque to failure was 24.3 ± 1.1 N m for the ABL constructs, 18.4 ± 2.0 N m for the ABN constructs, 20.5 ± 2.1 N m for the BN constructs and 19.2 ± 1.8 N m for the UL constructs (Fig. 3a).

#### Angular displacement at peak torque

The ABL construct had a significantly higher angular displacement at peak torque than the BN and ABN constructs (p ≤ 0.0070). The UL construct had a significantly higher angular displacement at peak torque than the ABN construct (p = 0.0225). No statistically significant difference was noted between the UL and BN constructs (p > 0.60). Additionally, no significant difference was noted between the ABL and UL constructs (p > 0.13) in angular displacement at peak torque. The mean ± SD angular displacement at peak torque was 42.8° ± 8.4° for the ABL constructs, 25.2° ± 5.3° for the ABN constructs, 31.2° ± 7.5° for the BN constructs, and 35.3° ± 8.3° for the UL constructs (Fig. 3b).

#### Torsional stiffness

When comparing torsional stiffness, the ABN construct was significantly stiffer than the ABL and UL constructs (p ≤ 0.0073). No significant difference was detected between the ABN and BN constructs in torsional stiffness (p > 0.58). Additionally, no significant difference was detected between the ABL and BN constructs (p > 0.14). The UL construct had significantly less torsional stiffness than all other constructs (p ≤ 0.0041). The mean ± SD stiffness was 1.0 ± 0.1 N m/deg for the ABL constructs, 1.2 ± 0.1 N m/deg for the ABN constructs, 1.1 ± 0.1 N m/deg for the BN constructs, and 0.9 ± 0.1 N m/deg for the UL constructs (Fig. 3c).

#### Failure mode

All constructs failed by synthetic bone fracture (comminuted and non-comminuted spiral fractures) propagated through screw holes. No screws or plates broke during testing of any of the constructs. Plastic deformation of the plates and screws (twisted and bent) was readily more obvious in the constructs which contained locking screws (ABL, BN, UL) (Additional file 1).

### Discussion

Of particular interest was to assess the difference if any between a single unicortical locking screw (UL) and a bicortical nonlocking (BN) placed nearest the fracture gap. The BN ‘hybrid’ construct outperformed the UL construct in torsional stiffness but not in peak torque or angular displacement at failure. This is likely the result of bicortical screw engagement which anchors into two cortices when compared to the unicortical screw and the compression of the plate to bone caused by the nonlocking screw.

The ABN construct had the highest stiffness of all constructs but interestingly had the lowest peak torque to failure and lowest angular displacement at failure. This may be explained by the compressive forces generated by the conventional screws between the bone and plate during initial loading of the constructs [10, 11]. Constructs with nonlocking screws compressed the plate to the synthetic bone thereby decreasing the distance from the central axis and as a result torque is resisted by the plate, bone and screws as a complete unit. In contrast with a locking screw, as the screw is tightened and the head engages the threaded screw hole, no further compression is generated between the plate and bone and therefore the screw/bone interface are the main components subjected to the torsional forces. All locking constructs (ABL and UL) had less torsional stiffness than the constructs which contained at least one nonlocking screw (ABN and BN). These findings are in agreement with previous studies which have demonstrated trends toward superior torsional stiffness for hybrid constructs [3, 5, 6, 12].

### Conclusion

The nonlocking constructs had the highest stiffness but lowest peak torsional forces at failure. Construct stiffness was found to exist in a very narrow range of 0.9–1.2 N m/deg with standard deviations of 0.1 N m/deg in all cases. It is unclear how relevant these minor differences would be in a clinical situation. The results of this study provide supporting evidence for the use of bicortical nonlocking screws in hybrid constructs to increase torsional stiffness. However, based on the current findings which report only on torsional loading, we cannot conclude that one construct is clearly superior to the other.

## Limitations

The anisotropic properties of bone play an important role regarding stiffness particularly during cyclical loading. Because the main objective was to compare the performance of four different screw configurations tested in single cycle to failure, the synthetic bone model served as a validated substitution [13]. While there are also limitations to testing with cadaveric bone, comparisons between the two could help further support the current results. The design of this study used the axis of the synthetic bone cylinders as the axis of rotation which introduces a translational effect with the plate under load which may differ in vivo. The cylinder axis was chosen based on previous similar in vitro and ex vivo torsional studies which have also used the axis of the bone or cylinder as the center of rotation [9, 14–16]. Unconstrained torsional testing which allows 5 degrees of freedom during a torsional load has been suggested by some authors as a method of reducing the variability introduced by tension within specimens [17, 18]. While it may have less variability, it remains technically challenging to implement and is thus currently not a routine method of biomechanical testing [18]. Furthermore, this study evaluated constructs in only one mode of loading, torsion. Other modes of testing such as bending and compression are required for a more complete biomechanical analysis.

## Additional file

**Additional file 1.** Relative implant deformation. Photograph demonstrating the relative amounts of plate deformation. Tangential lines extending from the screw tips have been inserted for illustrative purposes. Note the marked amount of torsional deformation for the ABL construct compared to all other constructs.

## Abbreviations

LCP: locking compression plate
LC-DCP: limited contact dynamic compression plate
ABL: all bicortical locking
ABN: all bicortical nonlocking
BN: bicortical nonlocking
UL: unicortical locking
AO: Arbeitsgemeinschaft Osteosynthesefragen
ASIF: Association for the Study of Internal Fixation
SD: standard deviation
N m: Newton meter
N m/deg: Newton meter/degree
