# Supplementary figures and images for: In vitro biomechanical testing of the 3.5 mm LCP in torsion: a comparison of unicortical locking to bicortical nonlocking screws placed nearest the fracture gap
Source: BMC Res Notes. 2017 Dec 27;10:768. doi: 10.1186/s13104-017-3102-y (PMC5745636; doi:10.1186/s13104-017-3102-y)

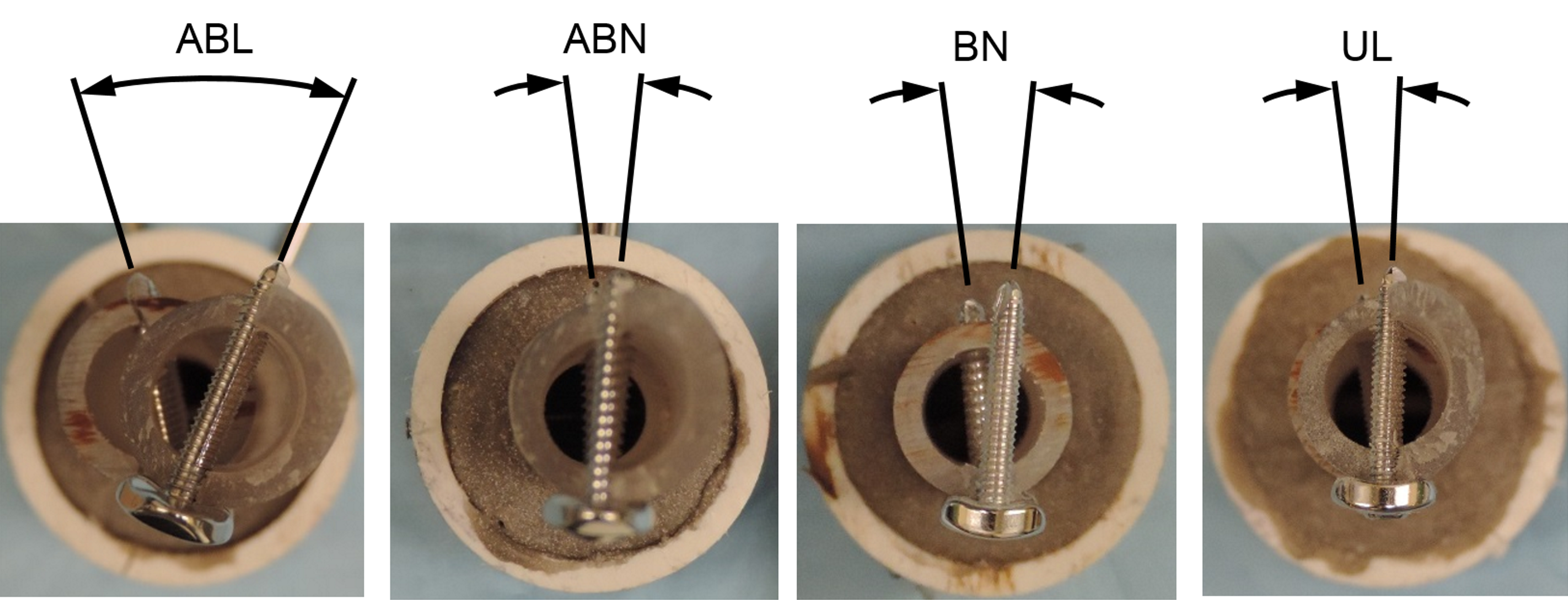

Supplement: Supplementary file 1 — Additional file 1. Relative implant deformation. Photograph demonstrating the relative amounts of plate deformation. Tangential lines extending from the screw tips have been inserted for illustrative purposes. Note the marked amount of torsional deformation for the ABL construct compared to all other constructs. [file 13104_2017_3102_MOESM1_ESM.tif]
